# Dependence of near‐surface similarity scaling on the anisotropy of atmospheric turbulence

**DOI:** 10.1002/qj.3224

**Published:** 2018-08-28

**Authors:** Ivana Stiperski, Marc Calaf

**Affiliations:** ^1^ Department of Atmospheric and Cryospheric Sciences, University of Innsbruck, Innsbruck, Austria; ^2^ Department of Mechanical Engineering, University of Utah, Salt Lake City, Utah

**Keywords:** anisotropy, boundary layer turbulence, CASES‐99, Reynolds stress tensor, similarity scaling

## Abstract

Turbulence data from the CASES‐99 field experiment, over comparatively horizontally homogeneous and flat terrain, are separated based on the anisotropy of the Reynolds stress tensor (into isotropic, two‐component axisymmetric and one‐component turbulence) and flux‐variance similarity scaling relations are tested. Results illustrate that different states of anisotropy correspond to different similarity relations, especially under unstable stratification. Experimental data with close to isotropic turbulence match similarity relationships well. On the other hand, very anisotropic turbulence deviates significantly from the traditional scaling relations. We examine in detail the characteristics of these states of anisotropy, identify conditions in which they occur and connect them with different governing parameters. The governing parameters of turbulence anisotropy are shown to be different for stable and unstable stratification, but are able to delineate clearly the conditions in which each of the anisotropy states occurs.

## 1 INTRODUCTION

Accurate numerical weather prediction is essential for many applications including transportation, agriculture, wind energy, hydrology and military (Katz and Murphy, 1997). For these applications, it is crucial that the region of the atmosphere closest to the land surface (atmospheric surface layer, ASL) is well captured numerically. Within the ASL, the land–atmosphere forcing is transmitted to the rest of the atmosphere via turbulent exchange of momentum, heat and moisture. The short time‐scales and limited spatial extent of these turbulent exchange processes make capturing them with traditional numerical weather prediction (NWP) models very difficult. NWP models, therefore, make use of similarity theory to compute surface fluxes of momentum, sensible and latent heat from the corresponding averaged quantities at a fixed height (*e.g.,*Brutsaert, 1982; Garratt, 1992; Wyngaard, 2010).

Strictly speaking, similarity relationships were originally developed for ensemble averages of statistically stationary and horizontally homogeneous surface layer flows (Monin and Yaglom, 1971). However, because it is in practice impossible to realize true ensemble averages in real field measurements, temporal averages are traditionally used, summoning the principle of ergodicity (*e.g.,*Katul *et al.* 2004; Wyngaard, 2010; Higgins *et al.* 2013). Furthermore, high‐resolution modern numerical methods such as Large Eddy Simulations (LES), rarely rely on the mean equations anymore but need detailed information about the fluxes as boundary conditions. To be able to model real flows over heterogeneous and complex surfaces, theory and applications must be reconciled under the principle of ‘*local*’ homogeneity and statistical stationarity. Meaning, that over small enough regions, sampled long enough, what *a‐priori* might resemble a heterogeneous surface, can ultimately be interpreted as homogeneous.

In this regard, the validity of surface layer similarity relations has been evaluated in terms of quasi‐steadiness and local homogeneity (*e.g.,*Brutsaert, 1982; Sugita and Brutsaert, 1990; Bou‐Zeid *et al.* 2005; Wyngaard, 2010; Hultmark *et al.* 2013; Babić *et al.* 2016a; 2016b), through numerical simulations (*e.g.,*Khanna and Brasseur, 1997; Bou‐Zeid *et al.* 2007; Stoll and Porté‐Agel, 2009) and data obtained in several experimental field campaigns located in places ranging from quasi‐perfect horizontal homogeneity (Kaimal and Finnigan, 1994) to highly complex terrain (*e.g.,*Martins *et al.* 2009; Nadeau *et al.* 2013; Stiperski and Rotach, 2016), and complex atmospheric conditions (*e.g.,*Grachev *et al.* 2013; 2016). From these results, procedures and rules‐of‐use have been developed to ensure appropriate use of similarity relationships.

Within this work, we present a new perspective on the range of validity of similarity scaling relations based on the anisotropy of the Reynolds stress tensor and the corresponding invariants (Lumley, 1978). In this novel approach, turbulence, instead of being separated into a coherent and a random part (cf. Salesky *et al.* 2017), is instead explored based on its topology. This topology, defined by the anisotropy, is then used as a means of assessing the success or failure of similarity relations. Note that similarity relations traditionally relate higher‐order moments (*e.g.,* fluxes of momentum and energy) that describe transport processes to lower‐order moments. In turbulent flows, it is the deviatoric or anisotropic portion of the Reynolds stress tensor (Pope, 2000) that is responsible for the turbulent transport of momentum and energy. Therefore it seems only natural to try to establish a relationship between similarity relations and turbulence anisotropy.

Lumley and Newman (1977) parametrized the Reynolds stress tensor $\bar{u_{i}^{\prime }u_{j}^{\prime }}$ in terms of the invariants of its anisotropy stress tensor, providing the possibility of quantifying variations of the stress tensor analytically. As proposed by Lumley, turbulence anisotropy can be used explicitly in the description of energy transfer, dissipation and turbulent transport (Jovanovic, 2004). In this regard, Banerjee *et al.* (2009) established a relationship between the dissipation tensor and the Reynolds stress tensor in axisymmetric turbulence. Klipp (2010a) used the CASES‐99 experimental data to investigate the statistical properties of the anisotropy stress tensor as a function of turbulence length‐scales. Results showed that the motions are near‐isotropic at the smallest scales, transitioning through pancake‐like axisymmetry at intermediate scales up to two‐dimensional large scales. In contrast, intermediate scales in urban canopies were found to be of the cigar type (Klipp, 2010b). In a more recent study, Klipp (2014) also used the anisotropic decomposition of the turbulence stress tensor as a means to provide new insight into the description of the outer length‐scale of the atmospheric boundary layer. As a result of this analysis, two new length‐scales were derived related to the transition between isotropic‐ and anisotropic‐type turbulence. One of these length‐scales was identified as a good candidate for the traditional definition of the outer scale used in optical applications.

The goal of this work is to re‐examine the near‐surface similarity scaling in light of turbulence anisotropy and to identify regimes in which different turbulent topologies are realized and how the transitions between anisotropic states are achieved. This novel approach could lead to improved surface‐layer parametrizations, as well as helping to advance numerical modelling of the land–atmosphere interface. For this purpose, we first focus on the bulk statistics of turbulence in the form of well‐known similarity scaling relations as functions of limiting states of anisotropy. We then isolate the governing parameters and identify the conditions under which these limiting states occur. Finally, we focus on the scale‐wise structure of turbulence and perform a spectral analysis of different turbulence topologies.

The article is organized as follows: in section 2 the invariant decomposition of the anisotropy stress tensor is reviewed and the dataset and post‐processing methods presented; section 3 presents the relationship between similarity scaling and turbulence anisotropy; section 4 identifies regimes and parameters governing anisotropy; section 5 examines the diurnal variation of the anisotropy and transitions between different limiting states; section 6 examines the spectral structure of turbulence anisotropy; an extended discussion of the results and implications for similarity theory is provided in section 7, with conclusions in section 8.

## 2 METHODOLOGY

### 2.1 Anisotropy of the Reynolds stress tensor

Turbulence is often described through the Reynolds stress tensor, expressed as

(1) $$\bar{u_{i}^{\prime }u_{j}^{\prime }}=\left(\begin{array}{ccc} \bar{u_{1}^{\prime }u_{1}^{\prime }}\;\;\; & \bar{u_{1}^{\prime }u_{2}^{\prime }}\;\;\; & \bar{u_{1}^{\prime }u_{3}^{\prime }}\\ \bar{u_{2}^{\prime }u_{1}^{\prime }}\;\;\; & \bar{u_{2}^{\prime }u_{2}^{\prime }}\;\;\; & \bar{u_{2}^{\prime }u_{3}^{\prime }}\\ \bar{u_{3}^{\prime }u_{1}^{\prime }}\;\;\; & \bar{u_{3}^{\prime }u_{2}^{\prime }}\;\;\; & \bar{u_{3}^{\prime }u_{3}^{\prime }} \end{array}\right),$$

where *ui′* denotes velocity fluctuations ($u_{i}^{\prime }=u_{i}-{\bar{u}}_{i}$) and the overline denotes the time‐averaging operation. Indices specify the rectangular Cartesian coordinates of different velocity components (*i* = 1, streamwise; *i* = 2, spanwise; *i* = 3 wall‐normal, i.e. vertical over flat terrain).

Since in turbulent flows, such as the atmospheric boundary layer (ABL), only the anisotropic components (*a*_*ij*_) of the Reynolds stress tensor are effective in transporting momentum (Pope, 2000), it is useful to distinguish between the isotropic and anisotropic contributions of the stress tensor. The isotropic stress is defined as $\frac{2}{3}\;e\delta_{ij}$, where *e* represents the turbulence kinetic energy ($e=1/2(\bar{u_{i}^{\prime }u_{i}^{\prime }})$). The components of the deviatoric, i.e. anisotropic, part are then defined as

(2) $$a_{ij}\equiv \bar{u_{i}u_{j}}-\frac{2}{3}e\delta_{ij},$$

which, upon normalization by 2*e*, results in a non‐dimensional anisotropy tensor with components

(3) $$b_{ij}=\frac{\bar{u_{i}u_{j}}}{\bar{u_{l}u_{l}}}-\frac{1}{3}\delta_{ij}.$$

As a result of this decomposition, it is possible to rewrite the Reynolds stress tensor in a simplified way, as the sum of the isotropic and anisotropic contribution $\bar{u_{i}u_{j}}=2e(\frac{1}{3}\delta_{ij}+b_{ij})$. To reduce the dimensionality of the problem further and therefore facilitate modelling and treatment of the anisotropic part of the stress tensor, Lumley and Newman (1977) identified two independent scalar invariants of the anisotropy tensor that describe the anisotropic part of the Reynolds stress tensor fully. Furthermore, the functional relationship between the invariants bounds the domain for all physically realizable turbulent flows (Jovanovic, 2004). In practice, this means that instead of six independent components of the stress tensor, the state of anisotropy can be described by two invariants, *η* and *ξ*, defined as 6*η* ^2^ = *b*_*ij*_ *b*_*ji*_ and 6*ξ* ^3^ = *b*_*ij*_ *b*_*jk*_ *b*_*ki*_, respectively (see Pope, 2000 for more details). The first invariant, *η*, is positive‐definite and measures the degree of anisotropy in the flow field (large values indicate large anisotropy and small values near‐isotropy). The second invariant, *ξ*, may instead be either positive or negative. For positive *ξ*, the flow is dominated by one‐component turbulence, while for negative values the flow is dominated by two‐component axisymmetric turbulence. The two scalar invariants of the anisotropy tensor can be derived alternatively through an eigenvalue decomposition of the anisotropy tensor, such that $\eta^{2}=\frac{1}{3}(\lambda_{I}^{2}+\lambda_{I}\lambda_{II}+\lambda_{II}^{2})$ and $\xi^{3}=-\frac{1}{2}\lambda_{I}\lambda_{II}(\lambda_{I}+\lambda_{II})$ (Spencer, 1971), where *λ*_*n*_ with *n* = *I*,*II*,*III* are the corresponding eigenvalues of the normalized Reynolds stress anisotropy tensor. As a result, turbulence can be categorized equivalently by the two invariants or by the eigenvalues of the anisotropy tensor, as shown in Table 1.

**Table 1:** Summary of special states of the Reynolds stress tensor in terms of the invariants (η, ξ) and eigenvalues of the anisotropy stress tensor as described by the Lumley triangle. The fourth column introduces the corresponding ellipsoid shape described by the eigenvectors ((Pope, 2000))

**Cases**	**Invariants**	**Eigenvalues**	**Shape ellipsoid**
Isotropic	*η* = *ξ* = 0	*λ*_*I*_ = *λ*_*II*_ = *λ*_*III*_ = 0	Sphere
Two‐component axisymmetric	$\eta =\frac{1}{6},\xi =-\frac{1}{6}$	$\lambda_{I}=\lambda_{II}=\frac{1}{6}$	Disk
One‐component	$\eta =\xi =\frac{1}{3}$	$\lambda_{I}=\frac{2}{3},\lambda_{II}=\lambda_{III}=-\frac{1}{3}$	Line
Axisymmetric, one large eigenvalue	*η* = *ξ*	$-\frac{1}{6}\leq \lambda_{II}=\lambda_{III}\leq 0$	Prolate spheroid
Axisymmetric, one small eigenvalue	*η* = −*ξ*	$0\leq \lambda_{I}=\lambda_{II}\leq \frac{1}{6}$	Oblate spheroid
Two‐component	$\eta ={\left(\frac{1}{27}+2\xi^{3}\right)}^{1/2}$	$\lambda_{I}+\lambda_{II}=\frac{1}{3}$	Ellipse

**Figure 1:**
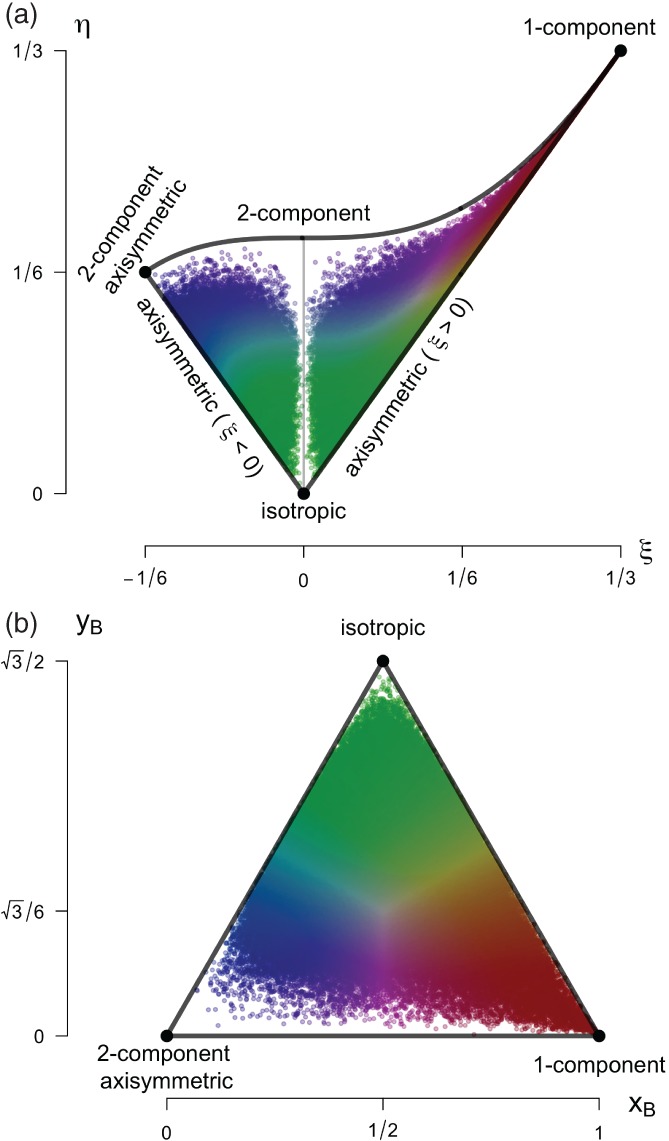
Anisotropy invariant maps: (a) Lumley triangle, function of the two anisotropy invariants η and ξ; (b) barycentric map, function of the anisotropy stress tensor eigenvalues and represented through the linearized coordinates x_B_ and y_B_. Data points correspond to the CASES‐99 dataset and are coloured based on their componentality according to Emory and Iaccarino (2014). Limiting states of the maps are specified.

Using this reduced set of variables, it is possible to depict the different states of turbulence graphically through the so‐called anisotropy invariant maps (AIMs: see Figure 1). When using *ξ* and *η* as independent variables, turbulence states are represented in the nonlinear Lumley triangle (Lumley and Newman, 1977; Choi and Lumley, 2001); alternatively, a barycentric map can be employed (Banerjee *et al.* 2007). This latter invariant map is a linear representation that weighs the different limiting states of turbulence anisotropy equally (Banerjee *et al.* 2007). It is spanned by a Euclidian domain, where the limiting states are placed at *x*_1*C*_ = (1,0), *x*_2*C*_ = (0,0) and $x_{3C}=(1/2,\sqrt{3}/2)$ and hence the coordinate system of the barycentric map (*x*_*B*_, *y*_*B*_) is defined such that

(4) $$\begin{array}{ll} x_{B} & =C_{1c}x_{1c}+C_{2c}x_{2c}+C_{3c}x_{3c}=C_{1c}+C_{3c}\frac{1}{2}, \end{array}$$

(5) $$\begin{array}{ll} y_{B} & =C_{1c}y_{1c}+C_{2c}y_{2c}+C_{3c}y_{3c}=C_{3c}\frac{\sqrt{3}}{2}, \end{array}$$

with the corresponding weights (*C*_*ic*_) determined by the eigenvalues of the normalized Reynolds stress anisotropy tensor, such that *C*_1*c*_ = *λ*_*I*_ − *λ*_*II*_, *C*_2*c*_ = 2(*λ*_*II*_ − *λ*_*III*_) and *C*_3*c*_ = 3*λ*_*III*_ + 1 (see Figure 1).

To facilitate one‐to‐one correspondence between turbulence data and information on the anisotropy, in this work we further use the RGB colour map of Emory and Iaccarino (2014) with adjustment to HSV colour space for visual purposes. This simple construction assigns colours to componentality behaviours. In this respect, one‐component turbulence is red, two‐component axisymmetric is blue and isotropic turbulence is green. All other states within the map are combinations of these colours (Emory and Iaccarino, 2014). We will use the colour‐map representation of the AIMs to classify the ABL turbulent flow and establish a relationship between traditional similarity relations and turbulence topology.

Note that the anisotropy invariants of the normalized Reynolds stress tensor, the magnitude of which is represented by an RGB combination, do not define the shape of any particular coherent turbulent structure, but rather provide a description of the eigenvalues of the stress tensor (Simonsen and Krogstad, 2005).

### 2.2 Dataset and data treatment

The Cooperative Atmosphere–Surface Exchange Study 1999 (CASES‐99) dataset (Poulos *et al.* 2002) forms the basis for our investigation. This well‐established dataset over horizontally homogeneous and semi‐flat terrain has already been used for testing and validation of similarity relations under mostly stable atmospheric stratification (*e.g.,*Klipp and Mahrt, 2004; Ha *et al.* 2007; Sorbjan and Grachev, 2010) among other purposes (*e*.*g*., Banta *et al.* 2002; de Wiel *et al.* 2003; Kumar *et al.* 2010; Mahrt, 2010; Sun *et al.* 2012; 2015; Sharma *et al.* 2017). The data consist of a month of measurements at a 60 m tower with seven levels of sonic anemometers (5, 10, 20, 30, 40, 50 and 55 m above ground). Two types of sonic anemometers were used in the study: levels 5, 30 and 50 m were installed with a non‐orthogonal CSAT3, whereas the other levels used orthogonal ATI‐K sonic anemometers. The data were processed using double rotation and were detrended prior to block averaging. Sensitivity tests have shown no great difference in the scaling results between data that have been detrended and those that have not, apart from a larger number of data fulfilling the stationarity criterion (see below).

Vertical gradients of potential temperature and wind speed needed for calculating the gradient Richardson number, $Ri=\frac{g}{\theta }\frac{\partial \theta }{\partial z}/{\left(\frac{\partial \bar{U}}{\partial z}\right)}^{2}$, were obtained by fitting an analytic profile to the data. For temperature, this profile was of the form $x=a+bz+cz^{2}+d\log(z)+e\log{\left(z\right)}^{2}$ and for wind speed $x=a+bz+cz^{2}+d\log(z)$. The turbulent kinetic enery (TKE) dissipation rate (*ϵ*) was calculated from the power spectra of the streamwise velocity component using the inertial dissipation method (cf. Piper and Lundquist, 2004). For this purpose, the inertial subrange was estimated to extend to *kz* = 1 following Katul *et al.* (2012). Here, $k=2\pi f/\bar{U}$ is the wavenumber, calculated from frequency *f* using Taylor's frozen turbulence hypothesis (Stull, 1988). The dissipation was only calculated for those data points that had a −5/3 slope in the inertial subrange (allowing a 10% error margin).

The data were not corrected for flux loss caused by sensor characteristics and sensor separation (Moore, 1986). The reason is that this frequency correction is developed mostly for the vertical components of the momentum flux and, if applied as such, would add asymmetry to the Reynolds stress tensor, causing unphysical anisotropy. Also, note that other studies of scaling using the CASES‐99 dataset report no use of flux corrections.

**Figure 2:**
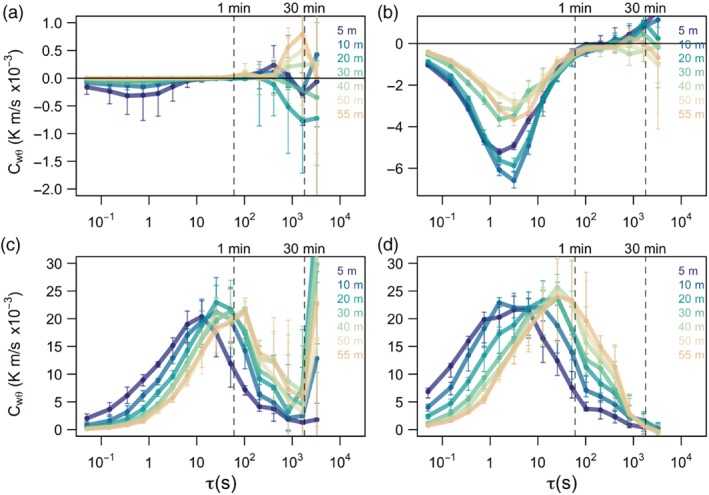
Multi‐resolution flux decomposition of heat flux for example periods with (a) strongly stable, (b) weakly stable, (c) strongly unstable and (d) weakly unstable stratification, respectively, for each height (shown in color). Vertical dashed lines indicate timescales of 1 min and 30 min. Error bars correspond to 25% and 75% percentiles.

Since the similarity scaling relations are only valid for turbulence, the multi‐resolution flux decomposition approach (MRD: *e.g.,*Howell and Mahrt, 1997; Vickers and Mahrt, 2003) was used to determine an appropriate averaging period needed to eliminate the contributions from non‐turbulent (sub‐)mesoscale motions, as well as to ensure the complete turbulent fluxes are accounted for (see Figure 2). This is achieved by examining the time‐scale at which the flux crosses over the zero line, which is usually considered the appropriate gap scale. For stable (night‐time) periods, MRD identified two limiting regimes that can be connected with strongly and weakly stable stratification. In the first, strongly stable regime (Figure 2a), the flux becomes zero at all heights at time‐scales of about one minute. The magnitude of the turbulent flux is also small, smaller than the (sub‐)mesoscale contributions at 30 min scales. In the weakly stable regime (Figure 2b), turbulence is more intense, has a larger time‐scale of around 5 min, and the (sub‐)mesoscale flux at 30 min is smaller than the turbulent flux. Given that these two regimes cross over the zero line at different time‐scales, as a compromise between the two regimes the most appropriate averaging time was chosen to be 1 min. Selecting an averaging time slightly lower than the limiting scale for the weakly stable case does not have a significant influence on the results.

Alternatively, for unstable (daytime) conditions the most appropriate averaging period was found to be 30 min (Figure 2c, d). As in the case of stable stratification, two distinct unstable regimes were identified: one having a substantial increase in mesoscale flux at periods larger than 30 min (Figure 2c), i.e. strongly unstable, and one for which these contributions are negligible (Figure 2d), i.e. weakly unstable. The results show that flux contributions by (sub‐)mesoscale motions can be as much as 99% in the strongly stable case and 65% in the strongly unstable case.

Prior to the analysis, all the data were subject to basic quality control (applying sonic flags and physical limits). These quality‐controlled data are shown in Figure 2c, d using the anisotropy classification introduced in section 2.1.

Since one of the objectives of this work is to increase our understanding of the relationship between turbulence anisotropy and similarity scaling, and given the prerequisites of similarity theory, data used for the scaling analysis were additionally required to satisfy the following quality criteria:

1. Night‐time unstable periods were discarded.
2. The gradient Richardson number had to be below the critical level, Ri < 0.25 (Grachev *et al.* 2013).
3. Stationarity of the data according to Foken and Wichura (1996) was required.

Stationarity was imposed on both the momentum and the sensible heat fluxes simultaneously. However, given the small values of momentum flux under convective conditions, stationarity of momentum flux was not imposed in this limit. Similarly, no stationarity of the sensible heat flux was required in near‐neutral conditions, where heat flux values are small.

The uncertainty criterion as described in Stiperski and Rotach (2016) was not applied to this dataset, given the short averaging time used for stable stratification, for which this criterion would eliminate all data. The omission of this criterion does not have any detrimental effects on the results for unstable stratification, where uncertain data are eliminated by the stationarity criterion.

**Figure 3:**
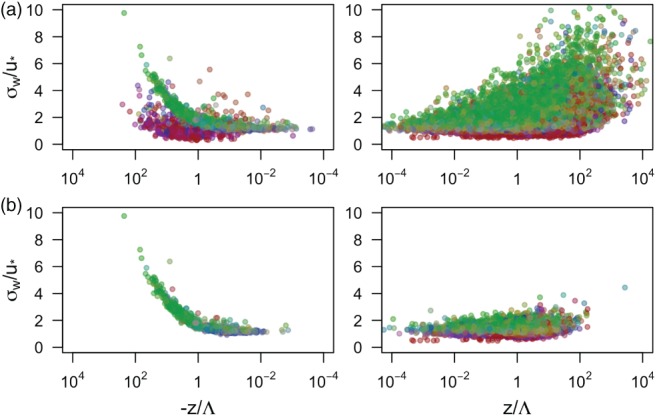
Projection of the data satisfying different quality criteria on the flux–variance relationship for the standard deviation of vertical velocity. (a) Data passing the basic quality control; (b) data that additionally satisfy criteria 1–3. Colour indicates the anisotropy state of the data: green for isotropic, blue for two‐component axisymmetric turbulence and red for one‐component turbulence.

The effects of imposing quality criteria 1–3 on the data are illustrated in Figure 3, where the data are coloured according to their anisotropy state (localized within the Lumley triangle or barycentric map from Figure 1). In the unstable regime, the additional information provided by the anisotropy classification illustrates the strong link between one‐component turbulence and “*low‐quality*” data, so that stationarity criterion practically eliminates the one‐component turbulence (Figure 3b). This type of turbulence can also be shown to correspond to night‐time countergradient fluxes. In the stable regime, however, the combination of stationarity criterion and Richardson number considerably reduces the number of data for all types of anisotropy, but does not eliminate all highly anisotropic data, so instances of stationary, Kolmogorov‐type (Ri < 0.25, cf. Grachev *et al.* 2013) one‐component turbulence are still encountered over the full range of *z*/Λ (see the definition in the next section).

Additionally, to facilitate interpretation of the results based on turbulence anisotropy, a fourth data‐quality criterion has been added. This is as follows:

4 Data falling within transition regions of the barycentric map, between different pure anisotropy states, were filtered out (see Figure 3).

**Figure 4:**
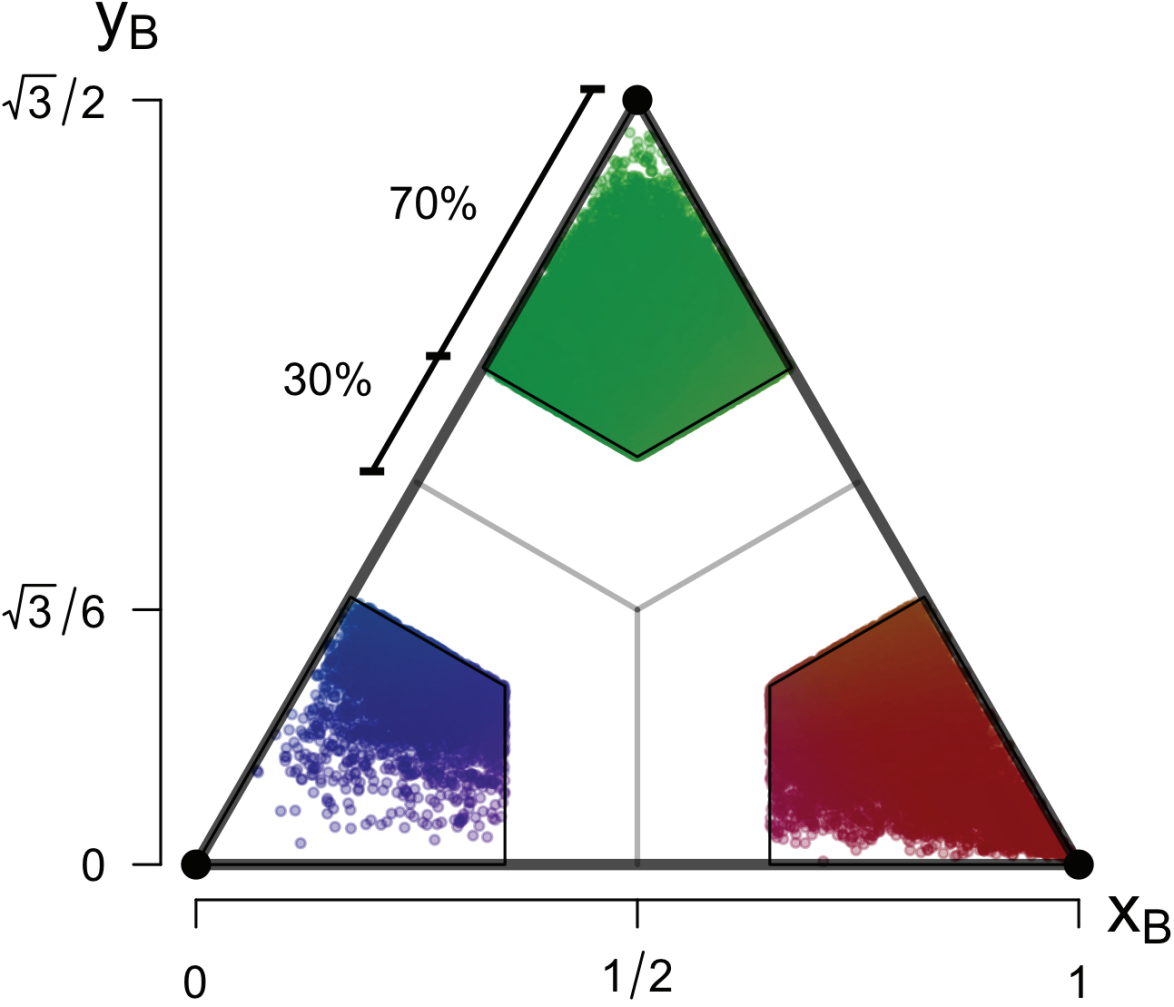
Barycentric map excluding the mixed states of anisotropy and hence only representing the the pure (i.e. limiting) anisotropic states.

This new data filtering process eliminates mixed states of anisotropy, reducing the focus of the analysis, in a first step, to the pure anisotropic states only (cf. Figure 4). The transition regions where these mixed states of anisotropy can be found were determined as those points falling outside the kite‐shaped regions of the barycentric map illustrated in Figure 4. The limiting lines for each kite were chosen to cover 70% of the sides of the equilateral triangle. Therefore only those data close to a pure anisotropic state are conserved for further scaling analysis. It is important to note that although, in a pure sense, the isotropic data are only those that sit on the vertex of the invariant map, in this article we will apply a less strict criterion than in e.g. Klipp (2010a) and refer to all data that fall within the green kite as isotropic and correspondingly for the other limiting states.

## 3 SCALING

The goal of this work is to provide a new perspective on near‐surface similarity scaling using additional information from turbulence anisotropy. For this purpose we employ the local scaling framework (cf. Nieuwstadt, 1984a; 1984b), in which all the turbulence quantities are computed and scaled at the corresponding measurement height *z*. This framework is more general than surface‐layer similarity theory, since it is applicable under both unstable and stable stratification. Under conditions in which the fluxes are expected to be constant with height (e.g. unstable stratification and near‐neutral stable stratification), local scaling should in principle correspond to surface‐layer scaling. For stable stratification, however, local scaling is more appropriate given the shallow nature of stable boundary layers, where higher tower levels are expected to be outside the surface layer. In the very stable limit, on the other hand, local scaling leads to *z*‐less scaling (cf. Sorbjan, 1987). In this respect, the local Obukhov length Λ is defined as $\Lambda =\frac{-u_{*}^{3}\theta_{v}}{\kappa g\bar{w^{\prime }\theta^{\prime }}}$, where *θ*_*v*_ is the mean virtual potential temperature and *κ*, the von Kármán constant, is taken as 0.4. The ratio *z*/Λ is therefore a measure of local stability (positive/negative values correspond to stable/unstable stratification at the measurement height). The local friction velocity is computed as $u_{*}={\left({\bar{u^{\prime }w^{\prime }}}^{2}+{\bar{v^{\prime }w^{\prime }}}^{2}\right)}^{1/4}$ and the local temperature scale as $\theta_{*}=-(\bar{w^{\prime }\theta^{\prime }})/u_{*}$.

As a reference, we use the well known surface‐layer flux‐variance similarity relationships for the standard deviations of velocity components (Φ_*u*_,Φ_*v*_,Φ_*w*_), following Panofsky and Dutton (1984):

(6) $$\Phi_{w}=\frac{\sigma_{w}}{u_{*}}=\left\{\begin{array}{c} 1.25{\left(1+3\frac{z}{\Lambda }\right)}^{1/3}\;\text{for}\;\frac{z}{\Lambda }>0,\\ 1.25{\left(1-3\frac{z}{\Lambda }\right)}^{1/3}\;\text{for}\;\frac{z}{\Lambda }<0, \end{array}\right.$$

(7) $$\Phi_{u,v}=\frac{\sigma_{u,v}}{u_{*}}=\left\{\begin{array}{c} 2.55{\left(1+3\frac{z}{\Lambda }\right)}^{1/3}\;\text{for}\;\frac{z}{\Lambda }>0,\\ 2.55{\left(1-3\frac{z}{\Lambda }\right)}^{\frac{1}{3}}\;\text{for}\;\frac{z}{\Lambda }<0, \end{array}\right.$$

for the standard deviation of temperature (Φ_*θ*_) following Tillman (1972) for unstable stratification and Pahlow *et al.* (2001) for stable stratification:

(8) $$\Phi_{\theta }=\frac{\sigma_{\theta }}{\theta_{*}}=\left\{\begin{array}{c} 3+0.05{\frac{z}{\Lambda }}^{-1}\;\text{for}\;\frac{z}{\Lambda }>0,\\ 0.95{\left(0.055-\frac{z}{\Lambda }\right)}^{1/3}\;\text{for}\;\frac{z}{\Lambda }<-0.05, \end{array}\right.$$

as well as the rate of turbulence dissipation (Φ_*ϵ*_) following Thiermann (1990):

(9) $$\Phi_{\epsilon }=\frac{\kappa z\epsilon }{u_{*}^{3}}=\left\{\begin{array}{c} {\left(1+4\frac{z}{\Lambda }+16{\left(\frac{z}{\Lambda }\right)}^{2}\right)}^{1/2}\;\text{for}\;\frac{z}{\Lambda }>0,\\ {\left(1-3\frac{z}{\Lambda }\right)}^{-1}-\frac{z}{\Lambda }\;\text{for}\;\frac{z}{\Lambda }<0.\; \end{array}\right.$$

Note that the form of the scaling relation for Φ_*u*_ and Φ_*v*_ used here corresponds to that for Φ_*w*_, however with a different near‐neutral limit (Stull, 1988). The neutral limit was chosen to be the same for both Φ_*u*_ and Φ_*v*_, despite the fact that the two are usually given different values (cf. Stull, 1988). The reasons for this will be shown later.

In the stable *z*‐less limit, (Sorbjan, 1987) suggested the following constant values of the flux–variance relationships:

(10) $$\Phi_{w}=1.6,\Phi_{u,v}=3.1,\Phi_{\theta }=2.4.$$

**Figure 5:**
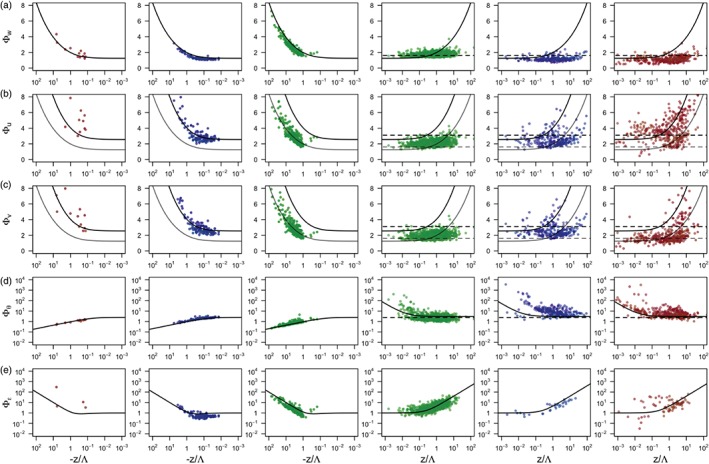
Flux–variance scaling relationships for the standard deviations of three velocity components: (a) Φ_w_,(b) Φ_u_ and (c) Φ_v_; (d) potential temperature, Φ_θ_, and (e) turbulence dissipation rate, Φ_ϵ_. From left to right, the data are separated according to the ABL thermal stratification (unstable for the three leftmost columns and stable for the three rightmost columns), as well as turbulence anisotropy (isotropic turbulence in green, one‐ and two‐component axisymmetric turbulence in red and blue, respectively). Data satisfy the quality criteria 1–4. Black solid lines correspond to scaling relations for each of the variables given in Equations (6), (7), (8), (9); black dashed lines correspond to the z‐less limit Equation 10. Grey lines in (b) and (c) are scaling lines for Φ_w_.

In order to examine the relationship between turbulence anisotropy and near‐surface scaling, Figure 5 illustrates the “*high‐quality*” data (those satisfying criteria 1–3) separated according to the three limiting states of anisotropy (criterion 4). In this figure, the three leftmost columns present the unstably stratified data and the three right most columns the stably stratified data. This representation allows an examination of the range of scaling relationships (Equations (6), (7), (8), (9)).The results show that the best correspondence between the scaling curves and the data is encountered for isotropic turbulence, under both unstable and stable stratification. This type of turbulence is also predominant in the high‐quality dataset (61% of unstable and 67% of stable cases).

In the case of unstable stratification, both the isotropic data (green) and two‐component axisymmetric data (blue) follow the traditional similarity relations (Equations (6), (7), (8), (9)) closely for commonly examined variables such as the standard deviation of vertical velocity (Φ_*w*_) and temperature (Φ_*θ*_), as well as the turbulent dissipation rate (Φ_*ϵ*_). The exceptions are the scaled horizontal velocity components (Φ_*u*_, Φ_*v*_). The reason for this is that for isotropic turbulence standard deviations of all three wind components have the same magnitude, therefore the scaled horizontal velocity components Φ_*u*_ and Φ_*v*_ follow the same similarity curve as Φ_*w*_ (Equation (6)), albeit with a somewhat larger scatter. This is also an indirect validation of the fact that the data are truly close to isotropic. In contrast, the scaled horizontal velocity components for the two‐component axisymmetric turbulence (blue) follow the scaling curves commonly applied to Φ_*u*_ (Equation (7)), while data for Φ_*v*_ do not suggest an alternative neutral limit, thus justifying our choice of the same scaling curves for Φ_*u*_ and Φ_*v*_. The fact that data for different limiting states of anisotropy (isotropic versus two‐component axisymmetric) follow different scaling curves for horizontal velocity components could explain the often encountered large scatter for these variables (cf. Banerjee *et al.* 2015), given that data with different kinds of anisotropy were thus far always examined together. It is also clear that these two limiting states of anisotropy occupy different stability regions. In contrast, the remnant one‐component turbulence (red) that was not eliminated by the quality criteria has large scatter and shows no apparent scaling of velocity components. This is turbulence that is aligned in the direction of the only eigenvector that has a corresponding non‐negligible eigenvalue. In terms of the Reynolds stress tensor, this turbulence state is characterized by negligible vertical velocity variance and therefore no longer corresponds to fully three‐dimensional turbulence. Hence this type of turbulence is bound to fail following any of the traditional scaling.

A very different behaviour is observed for stably stratified data. Under stable stratification, vertical turbulent motions are strongly attenuated by stratification, leading to small‐scale turbulence. The growing tendency of the flow to be decoupled from the surface with increasing stability results in *z*‐less scaling. This attenuation, however, affects different turbulence topologies in a different way. For isotropic turbulence, the attenuation of fluctuations is symmetric, so all the variables follow *z*‐less scaling (dashed lines in Figure 5) and, in analogy to unstable stratification, both of the horizontal velocity variances follow the scaling line for Φ_*w*_. On the other hand, for one‐ and two‐component axisymmetric turbulence, governed by multiple length‐scales, the attenuation by stratification is heterogeneous and vertical motions are more attenuated than horizontal ones. This causes only the vertical velocity variance Φ_*w*_ to follow what resembles *z*‐less scaling, although the limiting values are progressively lower than for isotropic turbulence. Φ_*ϵ*_ also fits the scaling line, but with larger scatter. All the other scaled variables (Φ_*u*_, Φ_*v*_, and Φ_*θ*_) exhibit significant scatter and a dependence on height (*z*/Λ) that deviates from *z*‐less scaling, but also do not appear to conform to any other well‐established scaling line. The reason that the one‐ and two‐component axisymmetric turbulence show larger dependence on *z*/Λ could be due to self‐correlation. Indeed, whereas the data that follow *z*‐less scaling are not correlated, a dependence of a variable on *z*/Λ imposed by *u*_∗_ featuring on both the *x*‐ and *y*‐axis will exhibit some level of self‐correlation. The standard approach of Klipp and Mahrt (2004) for estimating self‐correlation, however, fails for data that have a nonlinear relationship, such as the ones examined here, and is therefore not attempted here.

In traditional scaling studies, it is customary to observe large scatter for stably stratified data or for horizontal velocity variances in unstable stratification. With the proposed turbulence topology decomposition, based on the turbulence anisotropy, the reason behind this scatter becomes well understood. Note that, while traditional scaling relations are based on a single characteristic length‐scale, anisotropic turbulence can only be characterized by at least two different length‐scales. This should be taken into consideration when developing new similarity relationships to capture the turbulent fluxes of momentum, heat and energy better in the ABL. In addition, as mentioned earlier, similarity relationships were developed for idealized conditions of homogeneity and statistical stationarity and it has now long been observed that, under complex conditions (e.g. surface roughness and thermal heterogeneities, inclined slopes, etc.), these tend to fail and require additional corrections (e.g. Mironov and Sullivan, 2016). In view of the results presented in Figure 5, it is shown that an additional reason for the breakdown of traditional similarity relationships in complex conditions relates to the presence of strong turbulence anisotropy, with predominantly either one‐ or two‐ component turbulence. This will be studied in detail in a follow‐up article.

## 4 GOVERNING PARAMETERS

In the previous section, it was shown that all types of turbulence topology can be encountered for both stable and unstable stratification. The questions that arise at this point are whether these different states of anisotropy occur for the same reasons in both types of stratification and which parameters determine which type of turbulence topology will occur. The anisotropy analysis by itself is unable to provide an answer as to what processes generate each anisotropy state, as multiple processes can produce the same form of the Reynolds stress tensor. To isolate the governing parameters in stable stratification, we follow the hoceky‐stick transition (HOST) framework (Sun *et al.* 2015), which relates the evolution of the stably stratified boundary layer through the turbulence kinetic energy (TKE) and the mean wind speed $\left(\bar{U}=\sqrt{{\bar{u}}^{2}+{\bar{v}}^{2}+{\bar{w}}^{2}}\right)$. HOST identifies two distinct night‐time turbulence regimes: one governed by local shear and the other by global shear. Similarly, Salesky *et al.* (2017) have shown strong sensitivity of convective boundary‐layer characteristics to vertical wind shear ($\partial \bar{U}/\partial z$) and the local thermal stratification. To facilitate the connection between anisotropy states and governing parameters, quality criterion 4 is used to isolate limiting states of anisotropy. A unique averaging period of 30 min is used for both types of stratification in order to capture the overall characteristics of the environment, which for stable stratification also includes (sub‐)mesoscale contributions.

**Figure 6:**
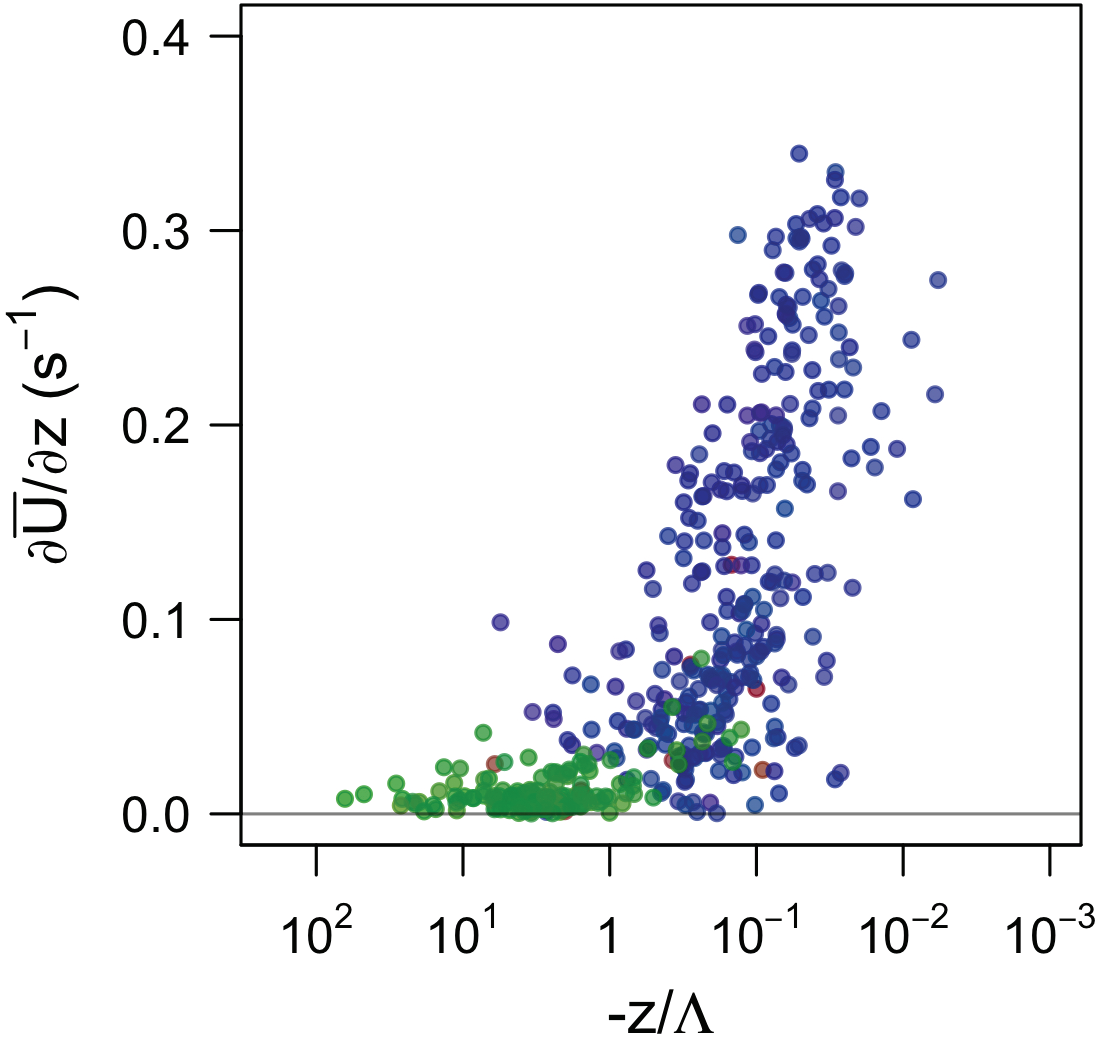
Characterization of turbulence anisotropy under unstable conditions by two characteristic parameters, wind shear $\partial \bar{U}/\partial z$ and stability z/Λ, for all measurement heights. Isotropic turbulence is shown in green and one‐ and two‐component axisymmetric turbulence in red and blue, respectively.

We first focus on the unstable stratification and examine the relationship between wind shear, local atmospheric stability (*z*/Λ) and turbulence geometry (Figure 6). Two distinct and almost non‐overlapping regimes can be isolated: isotropic turbulence exists only under conditions of very weak shear and very unstable (convective) stratification (−*z*/Λ > 1), regardless of the exact intensity of thermal instability. This corresponds to dynamic–convective and free‐convective sublayers according to Kader and Yaglom (1991). On the other hand, two‐component axisymmetric turbulence occurs solely under weakly unstable or near‐neutral conditions (0 < −*z*/Λ < 1) corresponding to the dynamic sublayer according to Kader and Yaglom (1991) and is correlated with increasing wind shear, although it is not dependent on the exact strength of the wind shear. Finally, the seldom encountered one‐component turbulence in unstable stratification shows no correlation with either thermal instability or wind shear, but is associated with wind‐speed profiles that vary significantly with height.

**Figure 7:**
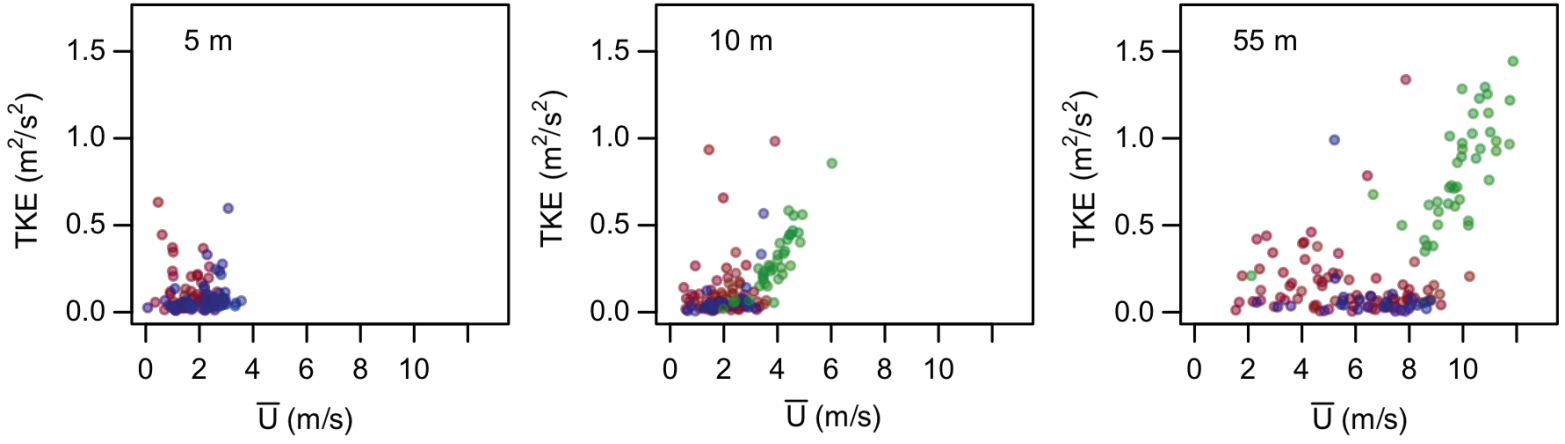
Characterization of turbulence anisotropy under stable conditions by two characteristic parameters, mean wind speed $\bar{U}$ and TKE, for three heights (5, 10 and 55 m). Isotropic turbulence is shown in green and one‐ and two‐component axisymmetric turbulence in red and blue, respectively.

The same governing parameters are unsuccessful in differentiating anisotropy types for stable stratification, since there, as shown in Figure 5, all types of anisotropy occur over the entire stability range (*z*/Λ). Here, we therefore employ the HOST framework (cf. Figure 7) instead and examine turbulence anisotropy as a function of turbulence kinetic energy and the mean flow velocity at different heights (shown here for 5, 10 and 55 m). Similarly to Sun *et al.*, 2012 (2012; 2015), we observe two turbulence regimes corresponding to different behaviours of turbulence with increasing wind speed. The present analysis shows that these two regimes are clearly separated according to turbulence anisotropy. The lower part of HOST for all examined heights corresponds to a mixture of one‐ and two‐component axisymmetric turbulence, for which TKE remains quasi‐invariant with mean wind speed. This branch corresponds to very stable conditions, where turbulence is often intermittent. The upper branch, on the other hand, is populated exclusively with isotropic turbulence and none of the other two limiting states. Given the lack of isotropy very close to the surface, we observe no upper branch at 5 m; however, mixed anisotropy states that were filtered out by the quality criterion 4 would still populate it. In a first approximation, one could conclude that the most relevant distinction in stable conditions is the one between isotropic and highly anisotropic turbulence. In order to distinguish between the occurrence of one‐ and two‐component axisymmetric turbulence in the very stable regime, however, it appears that alternative parameters beyond those provided by HOST or similarity theory are needed. This will be investigated in a future study.

It is interesting to note that this very clear correlation between anisotropy states and two regimes of HOST is lost when using the 1 min averages (not shown). In that case, HOST retains its form but isotropic turbulence is found in both regimes, mixed with other types of anisotropy. This shows that while for similarity theory it is vital to separate turbulence from non‐turbulent motions and therefore to use 1 min averages, the HOST framework is better suited for assessing the larger scale motions that characterize the night‐time environment and hence are better captured by 30 min averages. This result also confirms that, while the larger scale turbulence on very stable nights might be highly anisotropic, isotropic turbulence at very small scales can still be locally initiated.

## 5 DIURNAL EVOLUTION AND ROUTE TO ANISOTROPY

**Figure 8:**
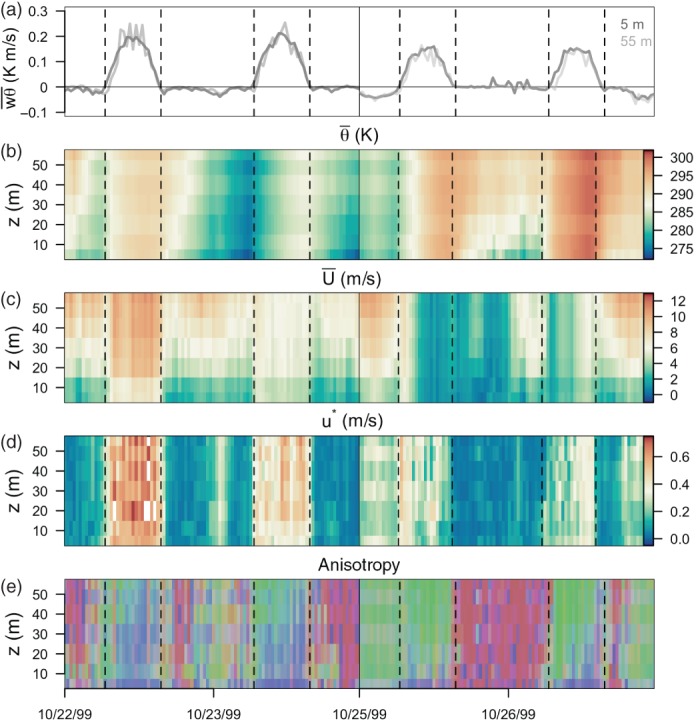
Daily evolution of (a) kinematic sensible heat flux, $\bar{w\theta }$, at 5 and 55 m height, (b) mean potential temperature, $\bar{\theta }$, (c) mean wind speed $\bar{U}$, (d) friction velocity, u_∗_, and (e) turbulence anisotropy coloured according to the barycentric map. Times are given in local time. Vertical dashed lines separate daytime and night‐time, determined as the times when the sensible heat flux changes sign. The vertical black line marks the time discontinuity between the night of October 23 and midnight of October 25.

In this section, the evolution of turbulence anisotropy is examined in the context of traditional ABL variables (mean temperature, mean wind speed, mean surface shear and sensible heat flux) for four quasi‐consecutive days (October 22, 23, 25 and 26; see Figure 8). For this analysis, we again use a unique 30 min averaging period for both unstable and stable stratification. Data quality control 1–4 (see Section 2.2) is also dropped, so as to avoid gaps in the daily evolution of the vertical profiles. In this way all ranges of anisotropy are represented. Therefore Figure 8 shows a clear continuous diurnal cycle of anisotropy. During the daytime, turbulence is predominantly two‐component axisymmetric (blue) or isotropic (green), while during the night‐time more mixed conditions are encountered, with the prevalence of one‐component (red) turbulence. From the contiguous vertical profiles, it is clear that close to the surface isotropy rarely occurs and turbulence is mostly two‐component axisymmetric, due to the presence of the wall and the importance of shear generation. Some exceptions to this rule occur during periods of stable stratification, when turbulence remains one‐component all the way down to 5 m. Daytime periods with low wind shear and modest values of friction velocity, corresponding to convective conditions (e.g. October 25 and 26), are well correlated, with close to isotropic turbulence already at 10 m above the surface, increasing with height, where it prevails throughout the day. Wind shear during the daytime causes turbulence to transition to two‐component axisymmetric. This transition is first initiated close to the surface and then diffuses towards higher altitudes (this is especially clear on October 23 and 26). At the same time, wind shear during the night‐time, coincident with a drop in the sensible heat flux, leads to close to isotropic turbulence (e.g. pre‐dawn on October 25). On nights with low forcing and strong inversion, one‐component turbulence prevails (e.g. the night of October 25/26). Persistent occurrence of two‐component axisymmetric turbulence during the night‐time is not encountered in the CASES‐99 dataset.

**Figure 9:**
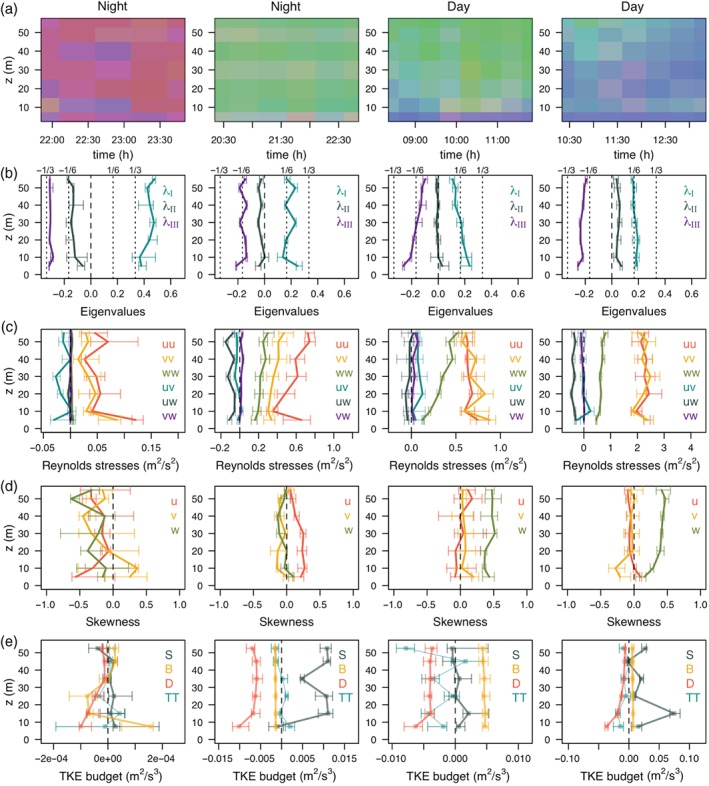
Example periods with strongly stable conditions and close to one‐component turbulence (first column; October 25), weakly stable conditions with close to isotropic turbulence (second column; October 27), convective conditions with isotropic turbulence (third column; October 25) and near‐neutral conditions with two‐component axisymmetric turbulence (fourth column; October 22). Shown are (a) anisotropy and profiles of (b) eigenvalues, (c) components of the Reynolds stress tensor, (d) skewness of velocity components and (e) TKE budget terms (B – buoyancy production/destruction, S – shear production, D – dissipation, TT – turbulence transport). Profiles present medians calculated over the time periods shown in (a), together with 25th and 75th percentiles.

In Figure 9, the influence of TKE generation and destruction mechanisms on anisotropy, as well as the characteristics of turbulence under these different regimes, are investigated more closely. Here, two characteristic night‐time (strongly and weakly stable) and daytime (convective and near‐neutral) periods, each consisting of two hours, are investigated in detail through vertical profiles of the eigenvalues (*λ*_*n*_, with *n* = *I*,*II*,*III*), Reynolds stresses ($\bar{u_{i}^{\prime }u_{j}^{\prime }}$, with *i*,*j* = 1,2,3), skewness ($(\bar{u_{i}^{{}^{\prime }3}})/{\left(\bar{u_{i}^{{}^{\prime }2}}\right)}^{3/2}$, with *i* = 1,2,3) and TKE budget terms.

The TKE budget equation used for this analysis is given by

(11) $$\frac{\partial e}{\partial t}+\bar{w}\frac{\partial e}{\partial z}=-\bar{u^{\prime }w^{\prime }}\frac{\partial \bar{U}}{\partial z}+\frac{g}{\bar{\theta }}\bar{w^{\prime }\theta^{\prime }}-\frac{\partial }{\partial z}\bar{w^{\prime }e^{\prime }}+\epsilon ,$$

where *e* is the TKE. Here, the TKE budget is only used to identify dominant terms, as well as their corresponding effect on turbulence anisotropy. Note that, given the one‐dimensionality of the measurements (i.e. measurements from only a single tower are used here), it is not possible to evaluate the full TKE budget closure (e.g. advection and horizontal shear production terms are not assessed). Instead, we focus only on the vertical terms and assume that turbulence is in a steady state, hence *∂e*/*∂t*≈0. This approximation, forced by the limitations of the tower measurements, is not that far‐fetched if one understands that the measurements were taken over fairly homogeneous terrain. The choice of double rotation, aligning the coordinate system with the mean wind direction at each height, used to post‐process the data means that the shear production term (*S*) is computed only through the contribution of $-\bar{u^{\prime }w^{\prime }}(\partial \bar{U}/\partial z)$. Also, because of the lack of horizontally distributed experimental data, only the vertical turbulent transport term of TKE can be computed ($TT=-\partial (\bar{w^{\prime }e^{\prime }})/\partial z$). The divergence terms were calculated using forward finite differences, whereas the respective fluxes were interpolated to the same height where divergence terms were given.

The profiles of eigenvalues and Reynolds stress components (Figure 9b, c) are provided as means to relate the turbulence anisotropy as observed from the principal axis frame of reference (or eigenvector space: Pope, 2000) and the standard streamwise coordinate system.

In strongly stable night‐time conditions, close to one‐component turbulence at 30 min averaging periods (Figure 9, first column) is associated with quite uniform vertical profiles of eigenvalues 1/3 ≤ *λ*_*I*_ ≤ 2/3, *λ*_*II*_∼ − 1/6, and *λ*_*III*_∼ − 1/3. These eigenvalues correspond to turbulence anisotropy ranging between one‐ and two‐component, or geometrically represented between an ellipse and a line (see Table 1). In streamwise coordinates, this type of turbulence is observed as having close to zero fluxes, a negligible vertical velocity variance and large horizontal velocity variances. As a result, motions are dominantly in the horizontal plane of the standard streamwise coordinate system, being hence quasi‐horizontally isotropic (*σ*_*u*_≈*σ*_*v*_) in this reference frame. It is of relevance to note that only at heights ∼10 m and 40 m where $\bar{u^{\prime }v^{\prime }}$ is zero, horizontal isotropy is achieved. It is also around these heights that turbulence is closer to being two‐component dominated (see Figures 9a, b). All the components of skewness show negative values indicating intermittency (cf. Kaimal and Finnigan, 1994). This type of turbulence occurs for weak wind conditions when turbulence destruction by stratification (B) significantly exceeds shear generation (S), and as seen in section 4 resides on the lower branch of HOST. Given the large non‐closure of the TKE budget for this type of condition, horizontal and non‐local contributions play a significant role.

As noted earlier, conditions closer to isotropy can only occur during nighttime in weakly stable conditions (Figure 9, second column) when the shear generation of turbulence is larger than buoyancy destruction. In the examined period, the eigenvalue profiles are once again uniform in height, with values of *λ*_*I*_∼1/6, *λ*_*II*_∼0, and *λ*_*III*_∼ − 1/6, illustrating that turbulence is actually a mixture of two‐ and three‐component turbulence states and is not axisymmetric. From the streamwise coordinates perspective the vertical and horizontal velocity variances are of the same order of magnitude. However, because the shear forcing is inhomogeneous, horizontal isotropy as represented in the streamwise coordinates is lost (Figure 9c). Unlike in the very stable case, vertical velocity has a Gaussian distribution (skewness = 0) in this regime. On the contrary, the streamwise component is positively skewed suggesting bursts of higher wind speeds (*i.e.,* gusts) embedded within a calmer environment. It is important to remember here again that the above analysis, suggesting lack of full isotropy (*σ*_*u*_≈*σ*_*v*_≈*σ*_*w*_) during nighttime, pertains to the 30 min averaging period.

The daytime periods are easily differentiated based on the dominant production terms in the TKE budget. For isotropic turbulence to occur it is necessary that buoyancy production (B) prevails over negligible shear generation (S) and that one is far enough from the wall. This is consistent with the free convective conditions encountered during the morning hours or generally under low synoptic forcing with weak winds. Turbulence becomes progressively more isotropic away from the surface in as much as the vertical velocity variance increases with height. The horizontal velocity variances, however, are constant throughout the tower depth. The approach to isotropy is also illustrated in the principal axis frame of reference through the trend of the eigenvalues with height. In this case, it is worth noting that all of the off‐diagonal terms of the Reynolds stress tensor are negligible, and hence both reference frames are most strongly aligned. Measurements from an even higher tower at Cabauw confirm that this kind of isotropic behaviour (*σ*_*u*_≈*σ*_*v*_≈*σ*_*w*_) extends well above 50 m (not shown). Vertical velocity has positive skewness (a sign of convection) for both the isotropic and two‐component axisymmetric regime. In the first case it is due to free convection, while in the second it is caused by forced convection when shear generation dominates the TKE budget. In the second, near‐neutral daytime case (Figure 9, fourth column) the horizontal velocity variances are significantly larger than the vertical velocity variance, leading towards two‐component axisymmetric behaviour as observed in the principal axis reference frame. There are indications that even under this regime, turbulence at higher altitudes might tend towards isotropy.

In both of these daytime regimes horizontal isotropy is preserved in the standard streamwise reference frame and explains the finding that both Φ_*u*_ and Φ_*v*_ have the same neutral limit (Equation (7)) as seen in section 3.

## 6 SPECTRAL ANALYSIS

Results in the previous three sections have highlighted the connection between turbulence topology and different ABL regimes, and as such have established a clear correlation between anisotropy and the success or failure of similarity theory. This connection can be further validated by examining the Fourier power spectra of the turbulent flow under different ABL thermal stratifications and for each limiting state of anisotropy. The spectra offer a scale‐wise perspective on the turbulence topology.

**Figure 10:**
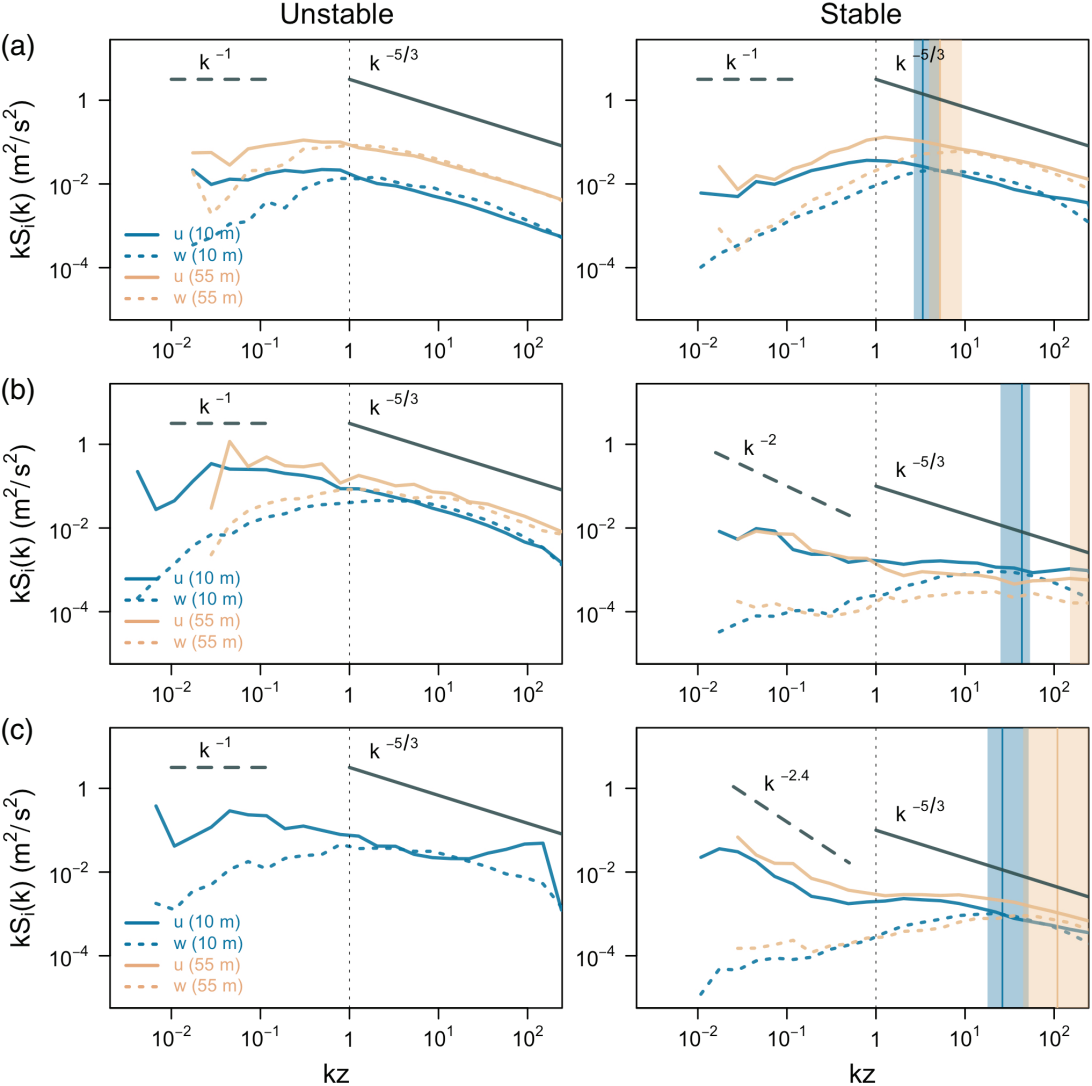
Median Fourier power spectra of the streamwise and vertical velocity components for two heights (10 and 55 m), computed over 30 min periods and averaged for all the limiting states of anisotropy: (a) isotropic, (b) two‐component axisymmetric and (c) one‐component turbulence, for unstable (left) and stable (right) stratification. Wavenumber k was calculated from frequency f using Taylor's frozen turbulence hypothesis and equals $k=2\pi f/\bar{U}$. Dashed vertical line shows kz = 1. Vertical solid lines correspond to median of the scaled Dougherty‐Ozmidov length scale z/L_oz_ for each height and the shading corresponds to the 25% and 75% percentiles.

Figure 10 shows the 30‐min median power spectra of the streamwise and vertical velocity components for each of the limiting states of anisotropy at two heights (10 and 55 m), as a function of *kz*, where *k* is the wavenumber. The medians were calculated from the scaled power spectra for each height by averaging over all instances of each of the three limiting states of anisotropy that were isolated using quality criterion 4 (see section 2). Prior to the spectral calculations, the signal was detrended. For the stable regime data the Dougherty‐Ozmidov length scale $L_{oz}=\sqrt{\epsilon_{w}/N^{3}}$ (*e.g.,*Dougherty, 1961; Ozmidov, 1965; Grachev *et al.* 2015), was also computed for each averaging period from the dissipation rate of the vertical velocity *ϵ*_*w*_, and the buoyancy frequency *N*, computed from the analytical profiles fit through the thermocouple data. The corresponding medians are represented with vertical lines and shaded regions are associated with the 25% and 75% percentiles.

The spectra for isotropic turbulence in both unstable and stable stratification (Figure 10a) show a well defined *k* ^−5/3^ slope, representative of the inertial subrange (Kaimal and Finnigan, 1994; Pope, 2000). For the unstable case, the −5/3 slope extends beyond the *kz* = 1 inertial limit (Katul *et al.* 2012), before transitioning to a *k* ^−1^ slope, representative of the TKE production subrange. This type of spectral extension of the −5/3 scaling had earlier been observed by Kader and Yaglom (1991), and is associated with the existence of free convection sublayer subjacent within the ABL's surface layer, influence of which was also noted in the previous section. The power spectrum of vertical velocity is identical to the streamwise component up until *kz* = 1, in line with the isotropic assumption. It, however, plateaus with a −1 slope to the left of the *kz* = 1 limit, quickly decaying in the smaller‐wavenumber regime. It is of relevance to note that in agreement with this spectral scaling (−5/3 and −1 scaling regimes) Katul *et al.* (2013), Banerjee *et al.* (2015) and Li *et al.*, 2015a (2015a; 2015b) were able to recover the traditional similarity scaling relations under the additional assumptions of spatial homogeneity and zero subsidence. Therefore the spectral analysis further justifies the success of similarity relations for isotropic turbulence illustrated in Figure 5. Also the two‐component axisymmetric turbulence (Figure 10b) under unstable conditions presents a well marked −5/3 slope for both the streamwise and vertical velocity components. However, in this turbulence topology, the −5/3 scaling for the vertical velocity component diverges almost a decade earlier in the wavenumber range than for the isotropic turbulence. In this regard, the spectral scaling of the vertical velocity component of the one‐component turbulence (Figure 10c) presents a very similar behavior. However, the streamwise velocity of the one‐component turbulence exhibits a quasi‐uniform −1 spectral slope throughout the wavenumber spectral range and large aliasing at high wavenumbers. The low number of cases with one‐component turbulence, however, does not allow a more thorough analysis of this regime.

Under stable stratification (Figure 10, right column), the difference between the spectra of isotropic turbulence and one‐ and two‐component axisymmetric turbulence is much more pronounced. For isotropic turbulence, both the streamwise and vertical velocity components show a well‐developed inertial subrange with a clear −5/3 slope. For the streamwise component, the inertial subrange extends almost to *kz* = 1, sharply decaying thereon without illustrating the existence of a production range. For the vertical velocity component, however, the inertial subrange covers a much narrower wavenumber range and the spectrum already starts to decay at *kz* = 10. Note that this scaled wavenumber qualitatively matches the Daugherty–Ozmidov length‐scale. On the other hand, spectra of both one‐ and two‐component axisymmetric turbulence show extensive similarities. In both, the strong stability suppresses turbulence, resulting in a very short inertial subrange, only found up to much larger wavenumbers. The onset of the inertial subrange once again matches the corresponding Daugherty–Ozmidov length‐scale, which is much lower than for isotropic turbulence. Note that in this case, however, the Daugherty–Ozmidov length‐scale presents larger deviations with height, a result of the fact that turbulence is more suppressed at higher levels. This coincidence of the Daugherty–Ozmidov length‐scale with the spectral transition of the inertial subrange seems to indicate that this length‐scale could be used to relate the nature of anisotropy to a turbulence length‐scale. This should be further explored in future works.

It is also interesting to note that in both cases a spectral slope of between −2 and −2.5 can be observed in the small‐wavenumber range. Similar spectral slopes can be a sign of two‐dimensional turbulence, as suggested by e.g. Lindborg (1999), Wyngaard (2010) and Lovejoy and Schertzer (2013). Alternatively, gravity waves (Dewan, 1997; Finnigan *et al.* 1984; Sukoriansky and Galperin, 2013), ubiquitous in very stable conditions, and Earth's rotation (Sukoriansky and Galperin, 2016) as well as buoyancy (Lovejoy and Schertzer, 2013) have also been invoked to explain spectral slopes ranging between *k* ^−2^ and *k* ^−3^. In all of these studies, however, these spectral slopes were observed in spectra obtained well above the boundary layer. It is safe to say that the low‐wavenumber part of the spectrum with a slope of about −2.5 corresponds to (sub‐)mesoscale motions, responsible for strong turbulence anisotropy. It is also worth noting that when a shorter averaging time is employed for the analysis (e.g. ∼1 min), this low‐wavenumber part of the spectrum is not taken into account. Given the possible existence of a −5/3 slope for individual spectra (although this is not necessarily observed on average), this shorter averaging time would lead to an improvement in the overall similarity scaling for stable conditions.

## 7 DISCUSSION

Results from this work show that turbulence reaches isotropy only away from the surface in weak wind conditions during the daytime, when buoyancy forcing dominates the TKE budget, and at smaller scales under strong wind conditions during the night‐time, when shear generation of TKE overpowers destruction by negative buoyancy. This antithesis of conditions leading to the generation of isotropic turbulence confirms that there is no single route to isotropy and that this route depends strongly on the ABL thermal stratification. Nonetheless, knowing the background thermal stratification (stable or unstable) and only two governing parameters ($\bar{U}$ and TKE for the stable cases and $\partial \bar{U}/\partial z$ and *z*/Λ for the unstable cases), turbulence topology associated with limiting states of turbulence anisotropy can be predicted successfully. Therefore, in conjunction with the scaling results from section 3, it should be possible to develop improved turbulence flux parametrizations. This is especially the case for horizontal variances, where relevant similarity relations are clearly stratified according to anisotropy. In a future extension of this work, one could also consider the relationship existing between surface boundary‐layer scaling and the three intrinsic directions of the Reynolds stress tensor.

It is also of relevance to note that the mere existence of close to isotropic turbulence states outside the inertial subrange in real near‐surface atmospheric conditions is in contradiction with the generally accepted premise that neither buoyancy nor shear can produce larger‐scale isotropic turbulence, due to the highly anisotropic nature of these forcings (cf. Wyngaard, 2010). Spectra for isotropic turbulence (Figure 10) have shown, however, that the existence of isotropy is related to the existence of a free convection sublayer within the ABL surface layer characterized by an extended −5/3 slope, as previously predicted by Kader and Yaglom (1991). The existence of isotropy is also in line with the existence of a single length‐scale in free convection (vertical only), as opposed to shear‐driven turbulence, which has two dominant length‐scales (vertical and horizontal) leading to two‐component axisymmetric behaviour.

Finally, the current analysis shows indications of sensitivity of the anisotropy analysis to the instrument used, under certain conditions. For example, during a weakly stable isotropic night (Figure 9, second column), the levels that have non‐orthogonal CSAT3 anemometers (i.e. 5 m, 30 m and 50 m) show less isotropic behaviour than levels where the measurements were done with orthogonal ATI‐K probes. This result is in line with recent findings (e.g., Frank *et al.* 2013; Horst *et al.* 2015) that non‐orthogonal sonic anemometers underestimate vertical motions (both variances and covariances) due to transducer shadowing. Such an underestimation would deform the Reynolds stress tensor and affect correct identification of flow anisotropy. Klipp (2010a) has also noted some sensitivity of her results to the instrument used. This finding, however, deserves further investigation.

## 8 CONCLUSIONS

In this work similarity relations were examined in the light of turbulence anisotropy. It was shown that, for the case of unstable stratification, both buoyancy‐driven isotropic turbulence and shear‐driven two‐component axisymmetric turbulence fit surface‐layer similarity relations well. The only differences are scaling relations for horizontal velocity components (Φ_*u*_, Φ_*v*_), which in the case of isotropic turbulence correspond to the similarity relation for the standard deviation of vertical velocity Φ_*w*_ (Equation (6)) and in the case of two‐component axisymmetric turbulence fit the Φ_*u*_ relation (Equation (7)). This difference explains the large scatter commonly found for Φ_*u*_ and Φ_*v*_ in various datasets (much more significant than for other variables), since data with different types of turbulent anisotropy are commonly examined together. This result also highlights the fact that there can therefore be no single scaling curve for both states of anisotropy, unless anisotropy is taken into account in the scaling itself. The fact that both Φ_*u*_ and Φ_*v*_ follow the same scaling curve can be attributed to horizontal isotropy in the streamwise coordinate system, found for both of these two limiting states of anisotropy under unstable stratification.

For stable stratification, both the isotropic turbulence found in weakly stable conditions and the two‐component axisymmetric turbulence agreed well with *z*‐less scaling behaviour, nonetheless with a different neutral limit. The large scatter in two‐component axisymmetric stable turbulence could be a result of the underestimation of vertical velocity variance in non‐orthogonal CSAT3 sonic anemometers due to transducer shadowing in weak wind conditions. On the other hand, one‐component turbulence in both stable and unstable stratification was characterized by large scatter and strong deviations from similarity scaling. This type of turbulence was shown to occur in strongly stable, weak wind conditions during the night‐time and transition periods during the daytime. Indications of similarity with 2D turbulence were found for cases of one‐component turbulence. These include negligible vertical velocity variance, large and horizontally isotropic horizontal velocity variances and a (close to) −3 spectral slope of the streamwise wind component spectrum found at low frequencies.

Governing parameters that allow differentiation of these limiting states of anisotropy were isolated. During unstable daytime periods, it was found that a balance between buoyancy (*z*/Λ) and wind shear ($\partial \bar{U}/\partial z$) predicts the shape of turbulence anisotropy well. If buoyancy dominates (−*z*/Λ > 1), turbulence tends towards an isotropic state, particularly away from the surface, whereas for shear‐dominated forced convection (0 < −*z*/Λ < 1) turbulence was found to be two‐component axisymmetric. During stable periods, a combination of turbulence kinetic energy (*e*) and wind speed ($\bar{U}$) within the HOST framework clearly delineates the one‐ and two‐component axisymmetric turbulence encountered in very stable conditions from the more isotropic turbulence in weakly stable conditions; however, it is unable to differentiate between the one‐ and two‐component axisymmetric motions themselves.

Finally, with knowledge of the ABL thermal stratification (stable or unstable) and governing parameters, it therefore becomes possible to predict turbulence topology and hence attribute scaling relationships correctly. We believe that this new framework will help provide a unifying approach to data from all kinds of complex surfaces. This new perspective on land–atmosphere turbulent exchange processes might also lead to improved parametrizations, as well as helping to advance numerical modelling of the land–atmosphere interface.

## ACKNOWLEDGEMENTS

We thank two anonymous reviewers for their positive remarks and useful comments. Mathias W. Rotach and Gabriel G. Katul are thanked for fruitful discussions, as well as Raul Bayoan Cal and Naseem Ali for their insight regarding the anisotropy of the Reynolds stress tensor. This research was funded by Austrian Science Fund (FWF) grant T781‐N32 awarded to Ivana Stiperski. Marc Calaf also acknowledges the Mechanical Engineering Department at University of Utah for start‐up funds.

The authors declare no conflict of interest.
